# Atypical appearance of hepatic hemangiomas with contrast-enhanced ultrasound

**DOI:** 10.18632/oncotarget.24185

**Published:** 2018-01-11

**Authors:** Min Huang, Qiyu Zhao, Fen Chen, Qihan You, Tian’an Jiang

**Affiliations:** ^1^ Department of Ultrasound, The First Affiliated Hospital, College of Medicine, Zhejiang University, Hangzhou, Zhejiang Province, China; ^2^ Department of Hepatobiliary Pancreatic Surgery, The First Affiliated Hospital, College of Medicine, Zhejiang University, Hangzhou, Zhejiang Province, China; ^3^ Department of Pathology, The First Affiliated Hospital, College of Medicine, Zhejiang University, Hangzhou, Zhejiang Province, China

**Keywords:** hepatic hemangioma, contrast-enhanced ultrasound, atypical

## Abstract

To describe the atypical enhancement pattern of hepatic hemangiomas on contrast-enhanced sonography. 22 patients with hepatic hemangiomas that were atypical on contrast-enhanced ultrasound were included in the study, all of them were confirmd by biopsy or surgery pathology. Atypical appearance of hepatic hemangiomas on contrast-enhanced ultrasound were divided into seven subtypes: (i) peripheral nodular enhancement in the arterial phase with centripedal filling, hypoechoic change in the delayed phase; (ii) peripheral circular enhancement; (iii) peripheral nodular enhancement; (iv) none enhancement; (v) septal enhancement; (vi) a central enhancing focus in the arterial phase, followed by a centrifugal enhancement in the portal venous phase, hypoechoic change in the delayed phase; (vii) slowly spoke wheel enhancement. Atypical appearance of hepatic hemangiomas were various. Radiologists should be aware. Establishing knowledge of the entire spectrum of atypical hepatic hemangiomas may benefit the rational approach to future cases.

## INTRODUCTION

Hemangioma is the most common benign tumor in liver, the prevalence varing from 1–2% [1] to 20% [2]. In grey scale ultrasound, hemangiomas typically appear as hyperechoic, well defined lesions, or hypoechoic masses with hyperechoic periphery [3, 4]. However, when the features of the lesion are atypical at conventional ultrasound (US), further investigation is required. Contrast-enhanced ultrasound (CEUS) has been proven to be a reliable method for the characterization of the focal liver lesions [5, 6], it is a sensitive and very specific method for the diagnosis of hemangiomas [7]. Based on the guidelines [8] and clinical experience, the typical CEUS features of hepatic hemangioma were peripheral nodular enhancement or circular enhancement in the arterial phase with centripedal filling, hyperechoic/ isoechoic change in the portal venous phase and late phase. Additional CEUS features were rapid centripetal enhancement in the arterial phase, hyperechoic/isoechoic change in the portal venous phase and late phase, usually seen in small lesions [8]. However, atypical imaging findings of hemangiomas may also occur even when contrast agents are administered, here we analyzed retrospectively the imaging features of 22 patients with hepatic hemangiomas that were atypical on CEUS.

## RESULTS

### Patients

The patients were 9 (40.9%) men and 13 (59.1%) women aged between 27 and 67 years old (mean ± standard deviation, 48.1 ± 9.3 years). Two patients had abdominal fullness or pain, the other twenty patients were asymptomatic, and the tumors were found incidentally on regular checkup or on imaging for other diseases. Four patients had multiple lesions, but each person included only one lesion that with pathological result in the study, the other eighteen patinets had solitary lesion. Pathologic specimens were obtained at surgery (hepatic resection, *n* = 5) or by percutaneous ultrasound-guided core needle biopsy (*n* = 17).

### Ultrasound and contrast-enhanced ultrasound

**Figure 1:**
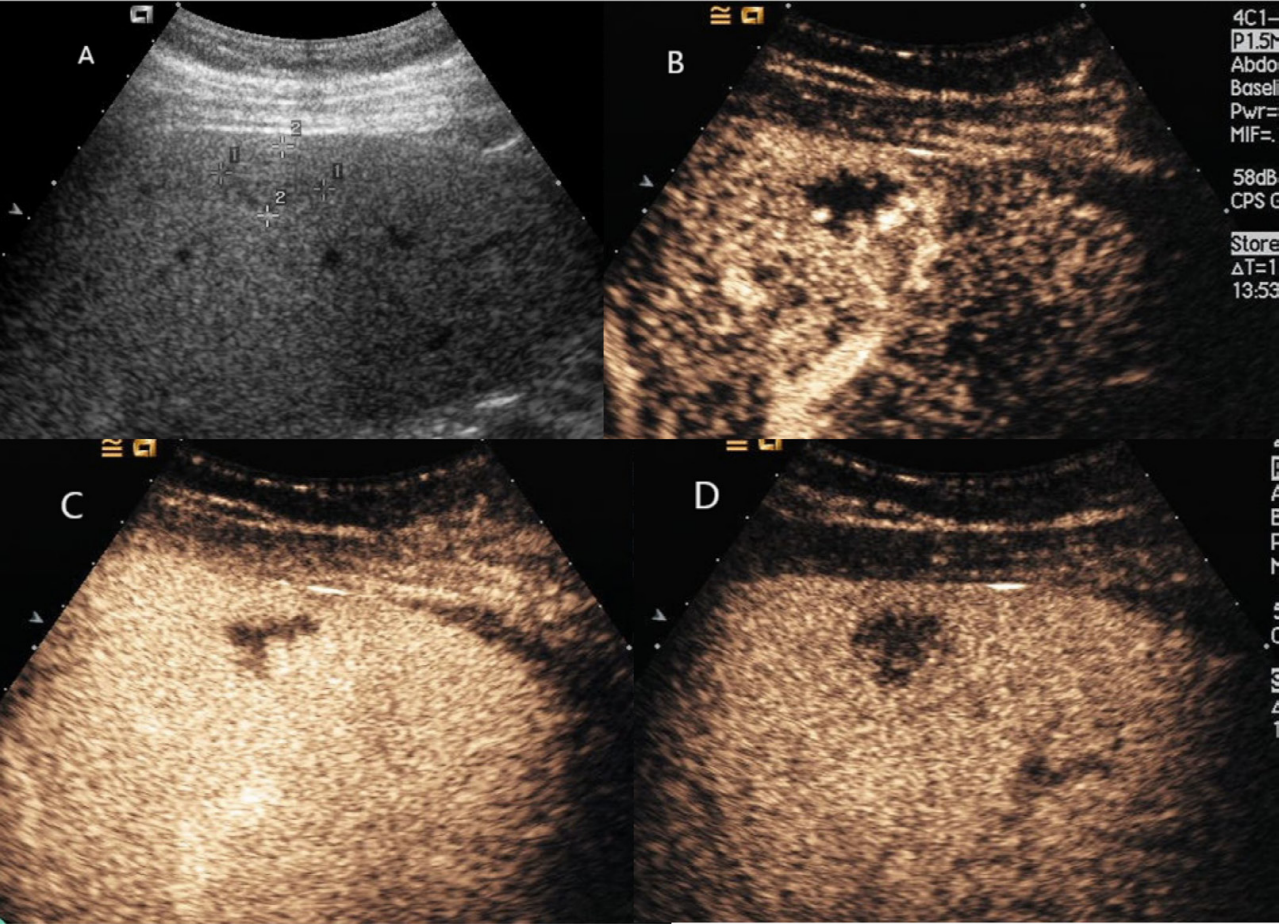
Hepatic hemangioma with washout in the delayed phase (**A**) Ultrasound revealed an isoechoic mass under right liver capsular. (**B**) and (**C**) CEUS showed peripheral nodular enhancement with centripedal filling. (**D**) CEUS showed hypoechoic change in the delayed phase.

**Figure 2:**
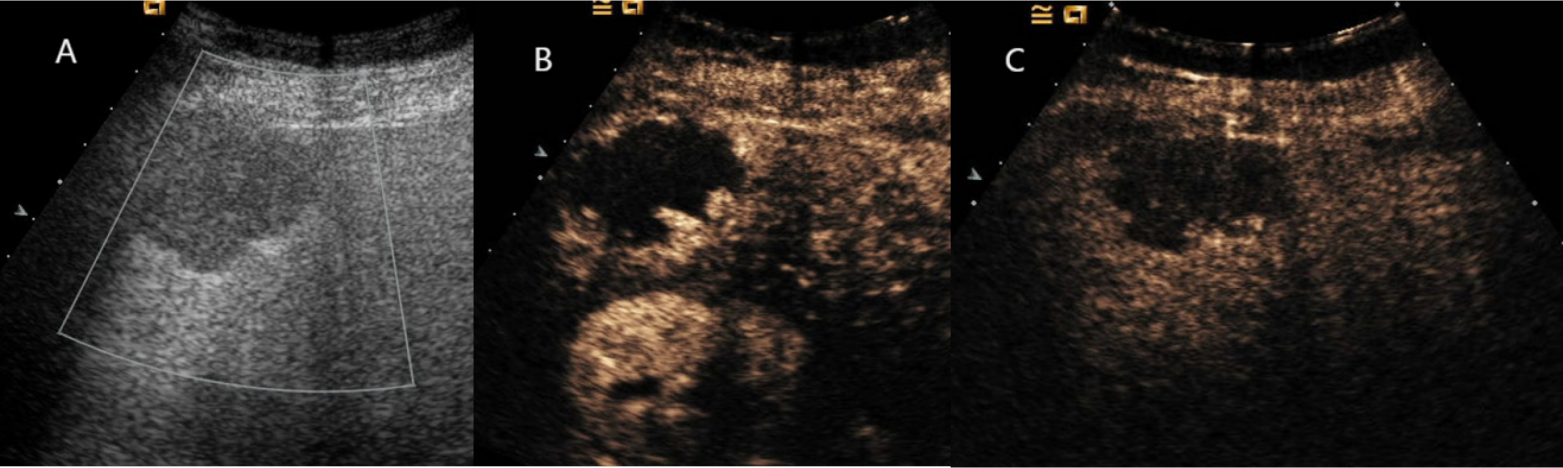
Hepatic hemangioma showed peripheral nodular enhancement in all vascular phases (**A**) Ultrasound revealed a hypoechoic mass under right liver capsular. (**B**) and (**C**) CEUS showed peripheral nodular enhancement throughout the whole vascular period.

**Figure 3:**
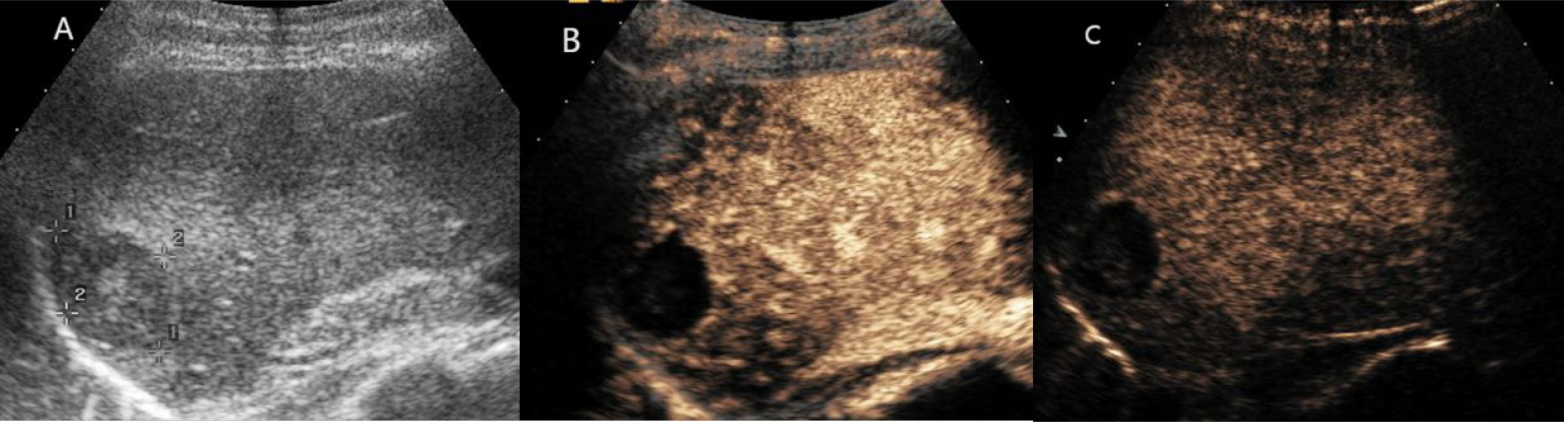
Hepatic hemangioma showed peripheral circular enhancement in all vascular phases (**A**) Ultrasound revealed a hypoechoic mass in the right liver. (**B**) and (**C**) CEUS showed peripheral circular enhancement throughout the whole vascular period.

**Figure 4:**
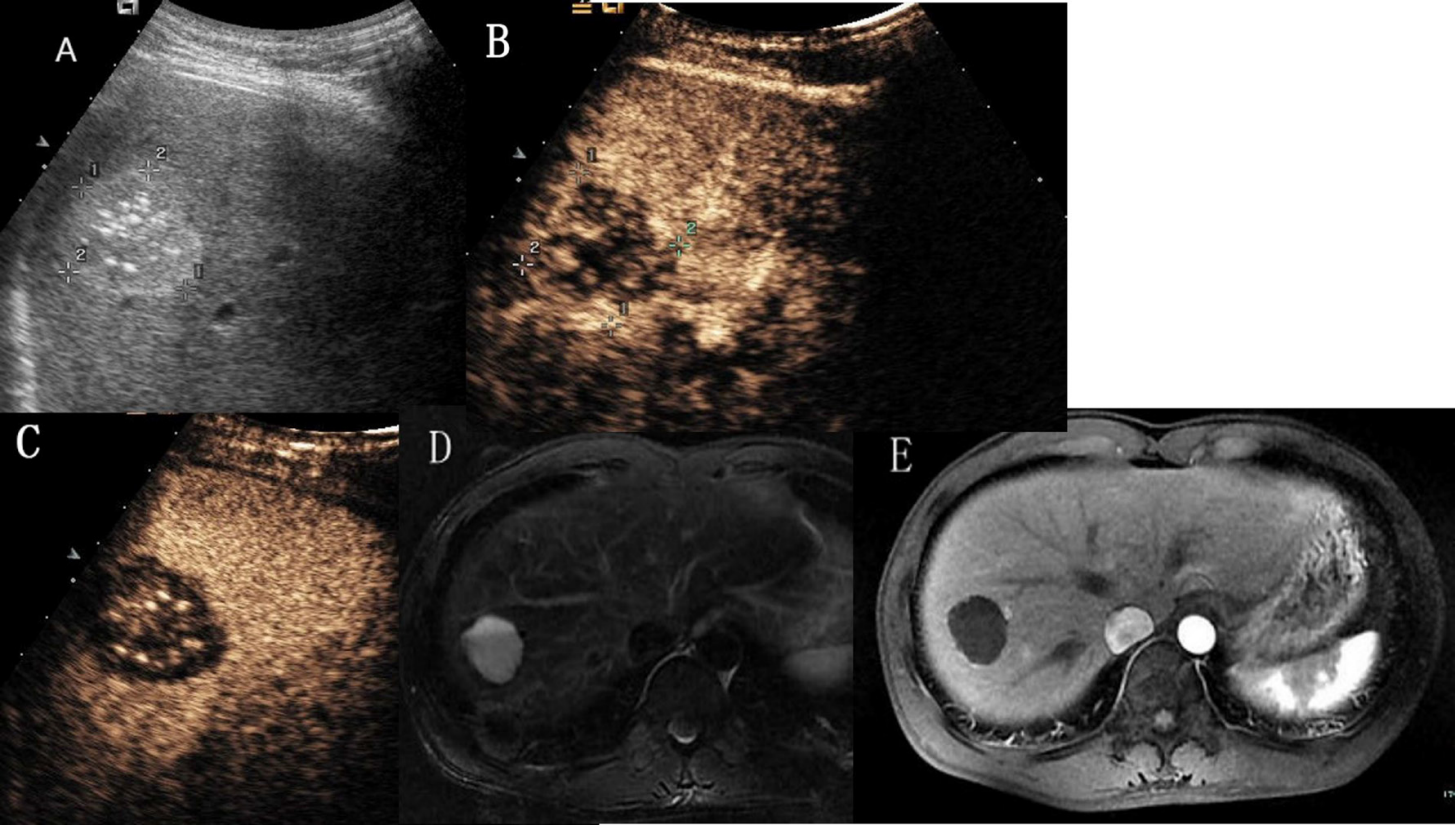
Hepatic hemangioma with no enhancement in all vascular phases (**A**) Ultrasound revealed a hyperechoic mass with multiple spotty calcifications in hepatic segment VII. (**B**) and (**C**) CEUS showed none enhancement throughout the whole vascular period. (**D**) T2- weighted MR images revealed a high signal intensity lesion with fluid–fluid level. (**E**) Enhanced magnetic resonance showed no contrast enhanced in arterial phase.

**Figure 5:**
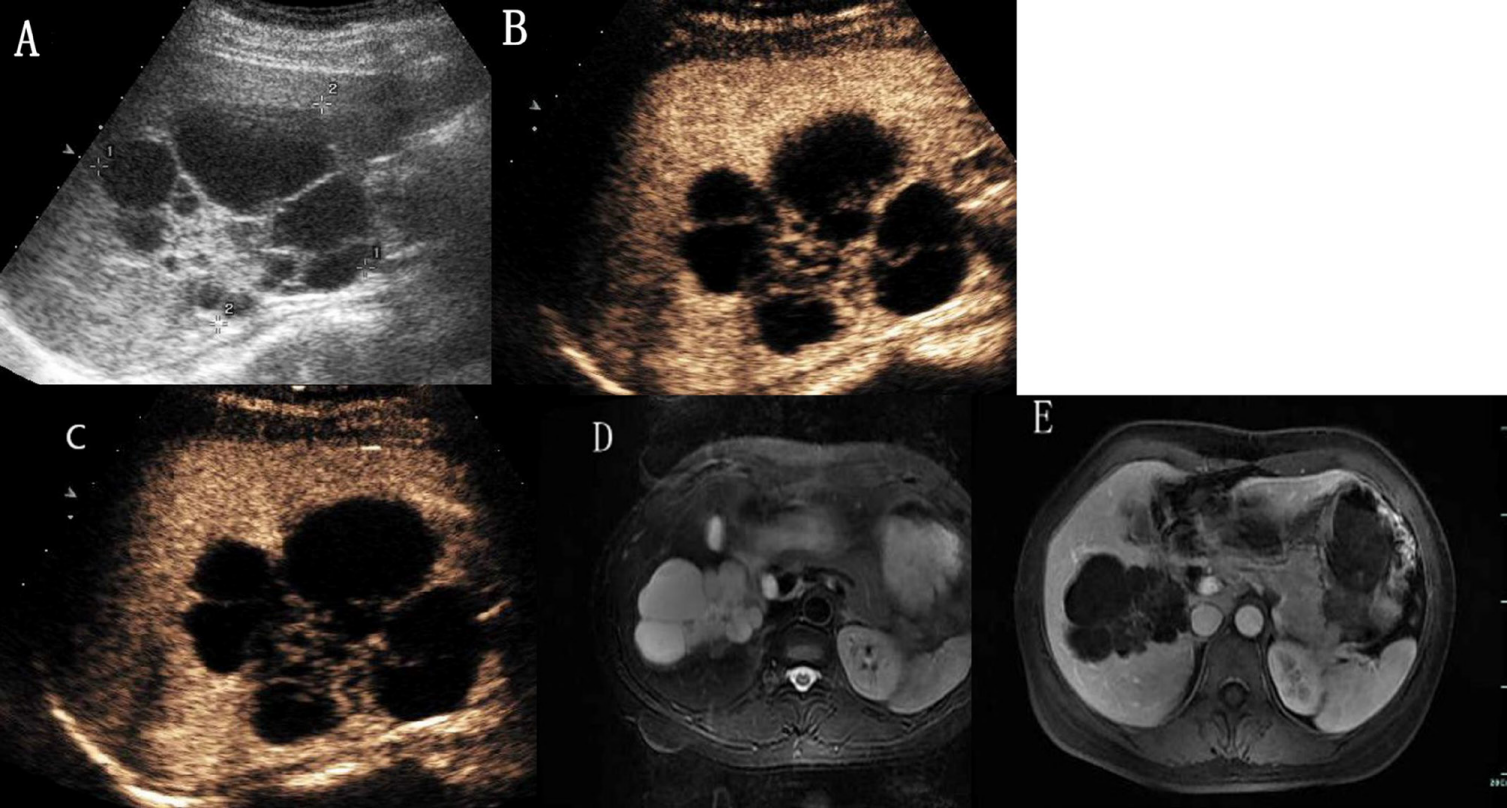
Hepatic hemangioma with septa enhancement in all vascular phases (**A**) Ultrasound revealed a mix-echoic mass comprised of a multilocular cystic part in the periphery and a stellate echogenic part in the centre. (**B**) and (**C**) CEUS showed isoenhanced with septa and stellate part, no enhancement with cystic part. (**D**) The lesion showed bright signal intensity on T2-weighted MR images. (**E**) On enhanced magnetic resonance, the septa part enhanced with contrast and the periphery cystic part was not enhanced.

**Figure 6:**
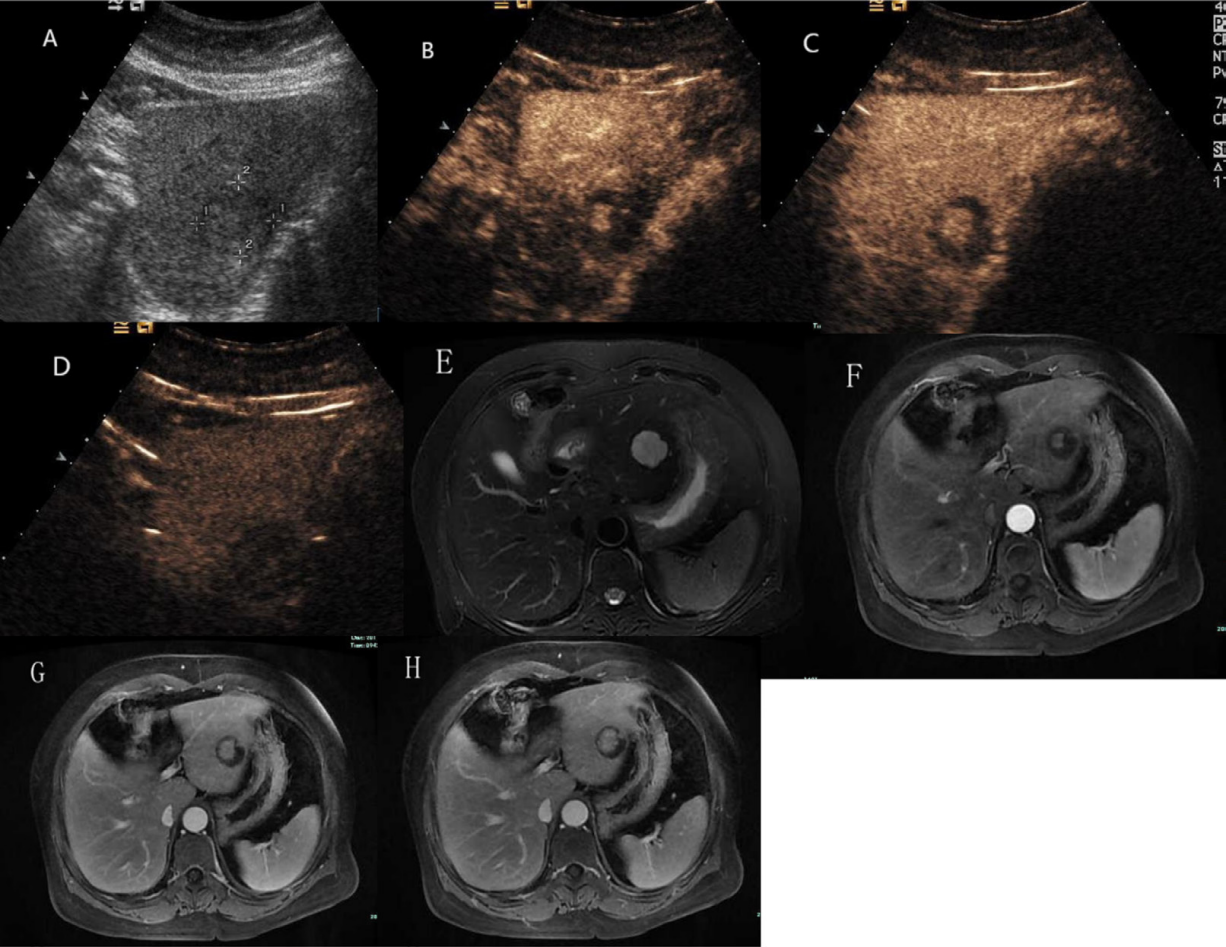
Hepatic hemangioma with centrifugal enhancement (**A**) Ultrasound revealed an isoechoic mass in the left liver. (**B**) and (**C**) CEUS showed a central enhancing foci in the arterial phase and followed by a centrifugal enhancement. (**D**) CEUS showed hypoechoic change in the delayed phase. (**E**) The lesion presented as markedly hyperintense on T2 weighted MR images. (**F**) Contrast enhanced MR images showed a central enhancing focus in the arterial phase. (**G**) and (**H**) The lesion showed centrifugal enhancement in the portal-venous phase and late phase.

**Figure 7:**
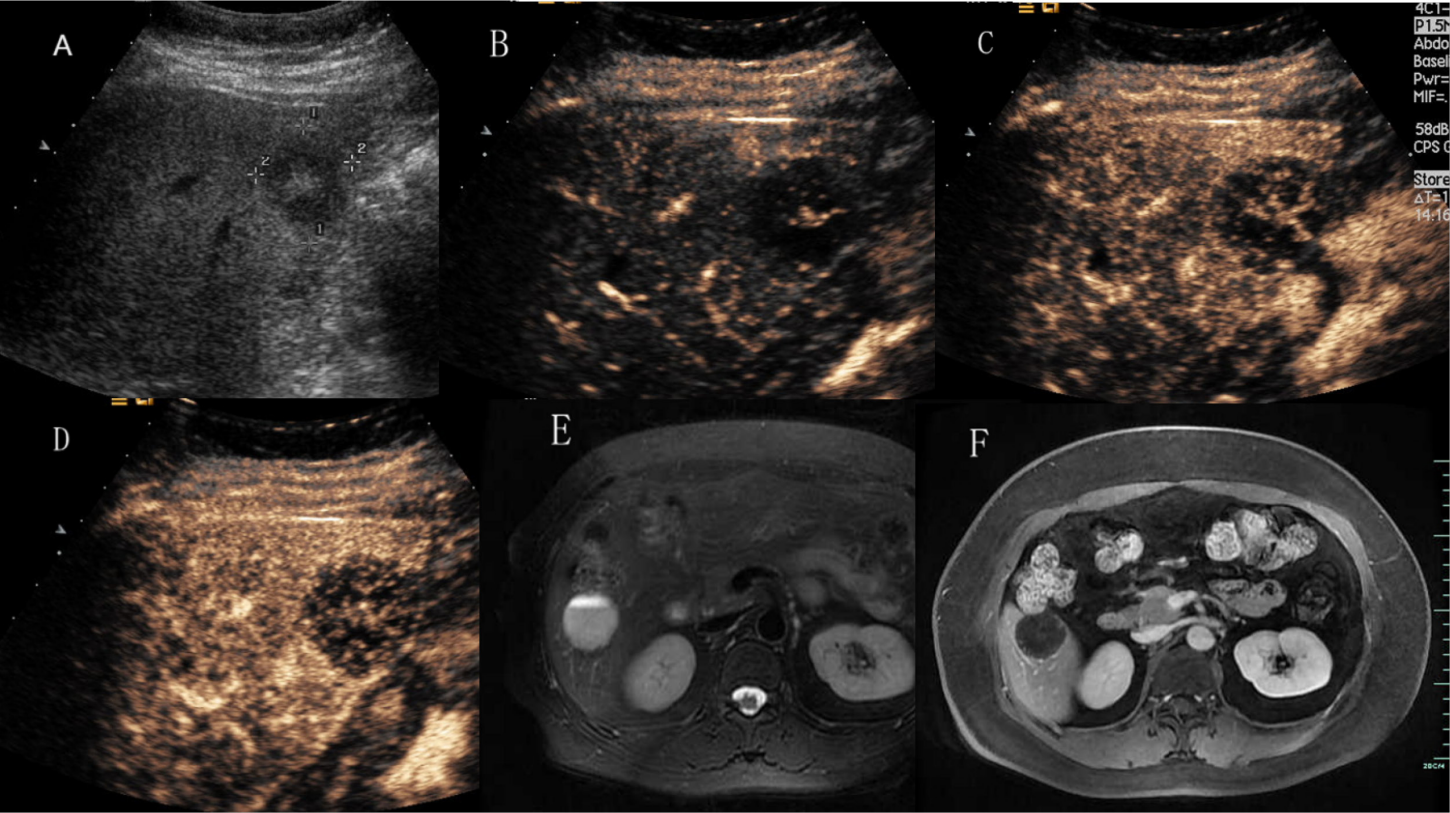
Hepatic hemangioma with slowly spoke wheel enhancement (**A**) Ultrasound revealed a hypoechoic mass in hepatic segment VI. (**B**), (**C**) and (**D**) CEUS showed slow spoke-wheel enhanced pattern. (**E**) T2-weighted MR images revealed a high signal intensity lesion with fluid–fluid level. (**F**) Contrast enhanced MR images showed slightly rim enhanced.

Of the 22 lesions, 18 (81.8%) had a clear border, 4 (18.2%) had an ill-defined border, 13 lesions were hypoechoic, 6 lesions were hyperechoic, 2 lesions were isoechoic, 1 lesion was multilocular cystic, 1 lesion had multiple calcifications. Peritumoral vascular signals were detected in 2 lesions. On the basis of ultrasound examination, 8 (36.4%) lesions were diagnosed as benign, 3 (13.6%) as malignant and 11 (50.0%) as uncertain, only 6 (27.3%) were diagnosed as hepatic hemangiomas. Atypical appearance of hepatic hemangiomas on CEUS were divided into seven subtypes: (i) peripheral nodular enhancement in the arterial phase with centripedal filling, hypoechoic change in the delayed phase (Figure 1); (ii) peripheral nodular enhancement throughout the whole enhancement period (Figure 2); (iii) peripheral circular enhancement throughout the whole enhancement period (Figure 3); (iv) none enhancement throughout the whole enhancement period (Figure 4); (v) septal enhancement throughout the whole enhancement period (Figure 5); (vi) a central enhancing focus in the arterial phase, followed by a centrifugal enhancement in the portal venous phase, hypoechoic change in the delayed phase (Figure 6); (vii) slowly spoke wheel enhancement throughout the whole enhancement period (Figure 7) (Table 1). On the basis of CEUS, 16 (72.3%) lesions were diagnosed as benign, 2 (9.1%) as malignant and 4 (18.2%) as uncertain, 13 (59.1%) were diagnosed as hepatic hemangiomas. Of the 22 patients, 18 underwent contrast-enhanced magnetic resonance (CEMR). On the basis of CEMR, 15 (83.3%) lesions were diagnosed as benign, 2 (11.1%) as malignant and 1 (5.6%) as uncertain, 12 (66.7%) were diagnosed as hepatic hemangiomas (Table 2).

**Table 1:** Ultrasound and contrast-enhanced ultrasound of 22 patients with hepatic hemangiomas

US					CEUS		
ID	Sex	Age (y)	Echogenicity	CDFI	Arterial	portal	late
1	M	40	hypo	No apprant	peripheral nodular	centripedal filling	hypoechoic
2	F	27	hypo	No apprant	peripheral nodular	centripedal filling	hypoechoic
3	F	39	hyper	No apprant	peripheral nodular	centripedal filling	hypoechoic
4	F	48	iso	Peritumoral vascular	peripheral nodular	centripedal filling	hypoechoic
5	M	55	hypo, posterior enhancement	No apprant	peripheral nodular	centripedal filling	hypoechoic
6	F	58	hypo	No apprant	peripheral nodular	centripedal filling	hypoechoic
7	M	57	hypo	No apprant	peripheral nodular	centripedal filling	hypoechoic
8	M	62	hypo	Peritumoral vascular	peripheral nodular	centripedal filling	hypoechoic
9	F	49	hyper	No apprant	peripheral nodular	centripedal filling	hypoechoic
10	M	67	hypo	No apprant	peripheral nodular	centripedal filling	hypoechoic
11	F	44	hypo	No apprant	peripheral nodular	centripedal filling	hypoechoic
12	F	46	hypo	No apprant	peripheral circular	peripheral circular	peripheral circular
13	M	39	hyper	No apprant	peripheral circular	peripheral circular	peripheral circular
14	F	41	hypo	No apprant	peripheral circular	peripheral circular	peripheral circular
15	F	51	hyper	No apprant	peripheral circular	peripheral circular	peripheral circular
16	M	47	hypo	No apprant	peripheral nodular	peripheral nodular	peripheral nodular
17	F	49	hyper	No apprant	peripheral nodular	peripheral nodular	peripheral nodular
18	M	59	hypo	No apprant	peripheral nodular	peripheral nodular	peripheral nodular
19	M	32	hyper, multiple calcifications	No apprant	no enhancement	no enhancement	no enhancement
20	F	48	multilocular cystic	No apprant	septal	septal	septal
21	F	50	iso	No apprant	a central enhancing focus	incomplete centrifugal	hypoechoic
22	F	51	hypo	No apprant	slowly spoke wheel	slowly spoke wheel	slowly spoke wheel

Note. CDFI = color Doppler flow imaging, CEUS = contrast-enhanced ultrasound, F = female, M = male, hyper = hyperechoic, hypo = hypoechoic, iso = isoechoic, US = ultrasound.

**Table 2:** Diagnostic accuracy of US, CEUS and CEMR for atypical hemangioma

Examination methods	Correct diagnosis	Misdiagnosis or undetermined	Diagnostic accuracy
US	6	16	27.3%
CEUS	13	9	59.1%
CEMR	12	6	66.7%

Note. US = ultrasound, CEUS = contrast-enhanced ultrasound, CEMR = contrast-enhanced magnetic resonance.

## DISCUSSION

Hepatic hemangiomas are being detected more frequently than before because of widespread application of routine ultrasound screening. Pathologically, hemangiomas are composed of many endothelium-lined vascular spaces separated by fibrous septa, and the collective size of their constituent vascular spaces may vary [9]. The differentiation of hemangiomas from other hepatic tumors is of great importance. CEUS has markedly improved the accurate diagnosis of hepatic hemangiomas, which is now possible in about 95% of cases [10]. It can provide diagnosis of hemangioma in most cases without requiring further investitation [11]. However, when the enhanced pattern was atypical, it may cause some uncertainty. Familiarity with the atypical appearance of hepatic hemangiomas on US and CEUS will increase the rate of correct primary diagnosis before surgery and histologic examination. In the present study, 11 hemangiomas showed washout in the delayed or portal phase, a common characteristic of them described in this series was their peripheral location on grayscale ultrasound, in one case near Glisson's capsule. Hemangiomas showed hypoenhanced compared to the adjacent liver parenchyma in the delayed phase, just like subtypes (i), were reported by several authors [12, 13, 14]. The mechanisms of washout had several hypotheses, most researchers [12, 13] believed that this probably occurred because of microbubble rupture due to prolonged insonation, which was not adequately compensated because of progressive hemodilution of contrast agent and a slow blood flow in these lesions. Most hemangiomas showed washout in the delayed phase were located in the liver sub-capsule, and some near Glisson's capsule [12, 14], we had the same situation in the study presented. Giannetti A *et al*. thought that hemangioms with arteriovenous and arteriosinusoidal shunts may washout in the early portal phase, which may have caused rapid elimination of contrast agent [14]. Hemangiomas with washout in the delayed or portal phase need differentiation from malignant liver tumors, especially metastasis. Bhayana *et al*. believed that the differential diagnosis between these lesions and metastases should be based on the time required for the elimination of microbubbles, which occurs much more rapidly in metastases [13]. During these 11 hemangiomas, 7 cases underwent contrast-enhanced magnetic resonance (CEMR), washout was not seen in all the cases. Magnetic resonance (MR) contrast agents had an extravascular equilibrium phase, and the interstitial portion of the hemangioma was filled during the delayed phase of the examination, which resulting in a hyper-isointense appearance compared with the adjacent parenchyma [15]. In our experience, hemangiomas with atypical appearance on CEUS due to washout may be diagnosed on the peripheral nodular enhancement and progression of centripedal filling in the arterial and portal phases of the examination. Besides washout in the late phase, the next most common atypical appearance of hepatic hemangiomas on CEUS was just peripheral enhancement without the progress of fill-in in all the vascular phases, just like subtypes (ii) and subtypes (iii). Since thrombo-haemorrhagic episodes, cystic degeneration, fibrosis or hyalinisation and calcium deposit may occur. Peripheral nodular enhancement was the typical feature of hemangioma in the arterial phase, if the peripheral nodular sustained hyper-isoechoic, it was helpful to make the diagnosis of hepatic hemangiomas. However, peripheral circular enhancement may cause some uncertainty, for it can be seen in other benign and malignant hepatic tumors, such as metastasis and inflammation. Without contrast enhancement throughout the whole enhancement period, just like subtypes (iv), is a very rare appearance of hemangioma, K. Mitsudo [16] reported a case of liver cavernous hemangioma which had multiple spotty calcifications arranged like a wreath and showed no enhancement on contrast-enhanced tomography. They misdiagnosed it as an old pyogenic abscess or cystadenoma preoperatively. Case 19 in our study was a 32-year-old man, he had a hyperechoic mass measuring 3.5 cm*3.1 cm in hepatic segment VII, which had multiple spotty calcifications and clear margin. Both CEUS and CEMR showed no contrast enhanced, on T2 weighted MR imaging, high signal intensity with fluid–fluid level could be seen (Figure 4). CEUS diagnosed it with old pyogenic abscess and CEMR diagnosed it with hepatic cyst. Core needle biopsy was performed and histopathologic examination revealed dialated blood vessels and collagen. The patient himself asked for surgery, so a partial hepatectomy was performed. The histological diagnosis was cavernous hemangioma with thrombosis and calcification. Hepatic tumors without contrast enhancement should not preclude the diagnosis of cavernous hemangioma. Multicystic appearance is very rare in atypical features of hepatic hemangiomas and several cases had been reported [17, 18, 19]. Case 20 in our study was a 48-year-old woman, she had a mix-echoic mass measuring 7.8*6.7*6.9 cm in the right liver. It was comprised of a multilocular cystic part in the periphery and a stellate echogenic part in the centre. On CEUS, septa and stellate part showed isoenhanced compared with the adjacent parenchyma, cystic part showed no enhancement through all vascular phases. On MR images, the lesion showed bright signal intensity on T2-weighted images, low signal intensity on T1-weighted images. Findings of CEMR was same as CEUS (Figure 5). Both CEMR and CEUS diagnosed it with biliary cystadenoma. The patient underwent partial hepatectomy and the histological diagnosis was multicystic cavernous hemangioma. The pathogenesis of the cystic change of hemangioma is not clearly understood, apoptosis had been proposed as one of the cause [20]. A predominantly cystic mass also should not preclude the diagnosis of cavernous hemangioma. Centrifugal (inside-out) enhancement was a more rare appearance of hepatic hemangioma. This enhancement pattern on dynamic contrast-enhanced computed tomography, CEMR and CEUS had also been reported [21, 22]. Case 21 in our study was a 50-year-old woman, she had a hypoechoic mass in the left liver measuring 2.9*2.7 cm. On CEUS, there was a central enhancing foci in the arterial phase and followed by a centrifugal enhancement in the portal-venous phase, it washed out in the late phase. The lesion presented as hypointense on unenhanced T1 weighted MR images and markedly hyperintense on T2 weighted MR images. CEMR confirmed the central enhancing focus in the arterial phase followed by a centrifugal enhancement in the portal-venous phase, but showed incomplete fill-in not wash-out in the late phase (Figure 6). Both CEUS and CEMR diagnosed it with hepatic carcinoma. The patient underwent partial hepatectomy and the histological diagnosis was cavernous hemangioma. This centrifugal (inside-out) enhancement should be differentiated from the central starlike fill-in enhancement which have a very high specificity for characterising focal nodular hyperplasia (FNH). Focal areas of intralesional enhancement were also observed in liver malignancies such as primary hepatic carcinoma and primary hepatic angiosarcoma [23]. In order to rule out malignancies, further investigation like core needle biopsy may be needed. Case 22 in our study was a 51-year-old woman, she had two hypoechoic masses in the liver, the larger one measuring 3.5*2.9 cm was located in hepatic segment VI, the other one measuring 3.3*2.8 cm was located in hepatic segment IV, on CEUS, the lesions showed very slow spoke-wheel pattern and were hypoenhanced throughout the vascular phases. On T2 weighted MR imaging, high signal intensity with fluid–fluid level could be seen, they presented as hypointense on unenhanced T1 weighted MR images, CEMR showed slightly rim enhanced in all vascular phases (Figure 7) and diagnosed it with metastasis.Core needle biopsy was performed in tumor that located in hepatic segment VI, and histological diagnosis was cavernous hemangioma. The patient had been followed up for more than two years. This slow spoke-wheel enhancement should also be differentiated from FNH which was always quickly enhanced and hyperechoic in arterial phase. Among the atypical appearance of hepatic hemangiomas, slow spoke-wheel pattern was extremely rare, there were few such reports available that describe this appearance.

## CONCLUSIONS

Atypical appearance of hepatic hemangiomas were various, including: washout in the late phase; just peripheral enhancement; no enhancement; septal enhancement; centrifugal enhancement and slow spoke-wheel enhancement. Radiologists should be aware of these rare atypical appearance. Establishing knowledge of the entire spectrum of atypical hepatic hemangiomas may benefit the rational approach to future cases.

## METHODS

### Patients

This study was approved by the ethics committee of the First Affiliated Hospital, College of Medicine, Zhejiang University, and informed consent was obtained from all patients. A retrospective analysis was performed of the records of all patients referred to our Institution between January 2007 and February 2016 who underwent CEUS for the assessment of focal liver lesions. Based on the literature [8] and clinical experience, typical CEUS findings of hepatic hemangiomas were classified into three categories: i) peripheral nodular enhancement in the arterial phase with centripedal filling, hyperechoic/isoechoic change in the portal venous phase and late phase; ii) peripheral circular enhancement in the arterial phase with centripedal filling, hyperechoic/isoechoic change in the portal venous phase and late phase; iii) rapid centripetal enhancement in the arterial phase, hyperechoic/isoechoic change in the portal venous phase and late phase, usually seen in small lesions. Other CEUS findings of hepatic hemangiomas were defined as atypical. We identified a total of 22 patients with hepatic hemangiomas that were atypical on CEUS, all of them were confirmd by biopsy or surgery pathology.

The machines was Acuson Sequoia 512 (Siemens Medical Solutions, Mountain View, CA) and LOGIC E9 (GE, Healthcare, Milwaukee, WI, USA) ultrasound system, which is capable of real-time contrast-enhanced imaging. The 3.5 MHz transducer was used with a mechanical index (MI) of 0.06–0.10. The contrast agent was SonoVue (Bracco, Milan, Italy), a sulfur hexafluoride-filled microbubble contrast agent, 2.4 mL was injected through a 20-gauge intravenous cannula into the antecubital vein, followed by a flush of 5 mL of 0.9% sodium chloride solution.

### US and CEUS examinations

US and CEUS examinations were performed by one of 3 experienced radiologists. The location, size, shape, echogenicity ,boundary and color of the lesion were recorded. Then the imaging mode was shifted to low-acoustic-power contrastspecific imaging. Low mechanical index values were used (from 0.06 to 0.10) in CEUS. The SonoVue was injected as described above. The timer was started promptly from the beginning of SonoVue administration, and the lesion was imaged in real time for 6 minutes, and the record was stored on the hard disk within the machine.

### Data analysis

All the ultrasound images and CEUS video clips were reviewed independently by three experienced radiologists blinded to the final diagnosis. They had at least 5 years of experience in liver CEUS interpretation. In case of inconsistent conclusions, a mutually accepted final conclusion was made via consultation. CEUS was evaluated during three phases as defined by Guidelines and Good Clinical Practice Recommendations for Contrast Enhanced Ultrasound (CEUS) in the liver ‒ Update 2012 [8]: the arterial phase (within 30 sec), portal venous phase (30–120 sec) and delayed phase (120–360 sec). The extent of enhancement of hepatic hemangiomas was referenced to the adjacent liver parenchyma and was divided into hyper-, iso-, hypo- and nonenhanced.
